# Supplementary material for: Novel Mutation in APC Gene Associated with Multiple Osteomas in a Family and Review of Genotype-Phenotype Correlations of Extracolonic Manifestations in Gardner Syndrome
Source: Diagnostics (Basel). 2021 Aug 28;11(9):1560. doi: 10.3390/diagnostics11091560 (PMC8466590; doi:10.3390/diagnostics11091560)
Supplement: Supplementary file 1 [file diagnostics-11-01560-s001.zip › diagnostics-1309091-SI.pdf]

Supplementary Materials

The gene panel used was Invitae Multi-Cancer panel (Invitae, San Francisco, CA). Full-gene sequencing, deletion/duplication analysis, and variant interpretation were performed at Invitae. The sequence analysis covers the coding exons and 10 to 20 base pairs of adjacent intronic sequence on either side of the coding exons. Genomic DNA obtained is enriched for targeted regions using a hybridization-based protocol, and sequenced using Illumina (Illumina, San Diego, CA). Reads are aligned to a reference sequence (GRCh37), and sequence changes are identified and interpreted in the context of a single clinically relevant transcript, indicated in the table (Table S1). All clinically significant observations are confirmed by orthogonal technologies, except individually validated variants and variants previously confirmed in a first degree relative. Confirmation technologies include any of the following: Sanger sequencing, Pacific Biosciences SMRT sequencing, MLPA, MLPA-seq, Array CGH. Array CGH confirmation of NGS CNV calling performed by Invitae Corporation (1400 16th Street, San Francisco, CA 94103, #05D2040778).

Table S1. Invitae Multi-Cancer panel (2019)

**Gene<br>(Transcript)**	**Gene<br>(Transcript)**	**Gene<br>(Transcript)**	**Gene<br>(Transcript)**	**Gene<br>(Transcript)**	**Gene<br>(Transcript)**	**Gene<br>(Transcript)**	**Gene<br>(Transcript)**
ABRAXAS1<br>(NM_139076.2)	***CDC73***<br>(NM_024529.4)	***EGLN1***<br>(NM_022051.2)	***FLCN***<br>(NM_144997.5)	***MLH1***<br>(NM_000249.3)	***POLE***<br>(NM_006231.3)	***RPL15***<br>(NM_002948.3)	***SLX4***<br>(NM_032444.2)
AIP<br>(NM_003977.3)	***CDH1***<br>(NM_004360.3)	***ENG***<br>(NM_000118.3)	***GALNT12***<br>(NM_024642.4)	***MLH3***<br>(NM_001040108.1)	***POT1***<br>(NM_015450.2)	***RPL19***<br>(NM_000981.3)	***SMAD4***<br>(NM_005359.5)
AKT1<br>(NM_005163.2)	***CDK4***<br>(NM_000075.3)	***EPCAM****<br>(NM_002354.2)	***GATA1***<br>(NM_002049.3)	***MRE11***<br>(NM_005591.3)	***PRKAR1A***<br>(NM_002734.4)	***RPL26***<br>(NM_000987.3)	***SMARCA4***<br>(NM_001128849.1)
ALK<br>(NM_004304.4)	***CDKN1B***<br>(NM_004064.4)	***ERCC4***<br>(NM_005236.2)	***GATA2***<br>(NM_032638.4)	***MSH2***<br>(NM_000251.2)	***PRSS1***<br>(NM_002769.4)	***RPL35A***<br>(NM_000996.2)	***SMARCB1***<br>(NM_003073.3)
AP2S1<br>(NM_004069.4)	***CDKN1C***<br>(NM_000076.2)	***EXT1***<br>(NM_000127.2)	***GEN1***<br>(NM_182625.3)	***MSH3***<br>(NM_002439.4)	***PTCH1***<br>(NM_000264.3)	***RPL5***<br>(NM_000969.3)	***SMARCE1***<br>(NM_003079.4)
APC<br>(NM_000038.5)	***CDKN2A***<br>*(p14ARF)*<br>(NM_058195.3)	***EXT2***<br>(NM_207122.1)	***GNA11***<br>(NM_002067.4)	***MSH6***<br>(NM_000179.2)	***PTCH2***<br>(NM_003738.4)	***RPS10***<br>(NM_001014.4)	***SPINK1***<br>(NM_003122.4)
ATM<br>(NM_000051.3)	***CDKN2A***<br>*(p16INK4a)*<br>(NM_000077.4)	***EZH2***<br>(NM_004456.4)	***GPC3***<br>(NM_004484.3)	***MUTYH***<br>(NM_001128425.1)	***PTEN***<br>(NM_000314.4)	***RPS19***<br>(NM_001022.3)	***STK11***<br>(NM_000455.4)
ATR<br>(NM_001184.3)	***CEBPA***<br>(NM_004364.4)	***FANCA***<br>(NM_000135.2)	***GREM1****<br>(NM_013372.6)	***NBN***<br>(NM_002485.4)	***RAD50***<br>(NM_005732.3)	***RPS20***<br>(NM_001023.3)	***SUFU***<br>(NM_016169.3)
AXIN2<br>(NM_004655.3)	***CEP57***<br>(NM_014679.4)	***FANCB***<br>(NM_001018113.1)	***HOXB13***<br>(NM_006361.5)	***NF1***<br>(NM_000267.3)	***RAD51C***<br>(NM_058216.2)	***RPS24***<br>(NM_033022.3)	***TERC***<br>(NR_001566.1)
BAP1<br>(NM_004656.3)	***CFTR***<br>(NM_000492.3)	***FANCC***<br>(NM_000136.2)	***HRAS***<br>(NM_005343.2)	***NF2***<br>(NM_000268.3)	***RAD51D***<br>(NM_002878.3)	***RPS26***<br>(NM_001029.3)	***TERT***<br>(NM_198253.2)
BARD1<br>(NM_000465.3)	***CHEK2***<br>(NM_007194.3)	***FANCD2****<br>(NM_033084.3)	***KIF1B***<br>(NM_015074.3)	***NTHL1***<br>(NM_002528.6)	***RB1***<br>(NM_000321.2)	***RPS29***<br>(NM_001032.4)	***TMEM127***<br>(NM_017849.3)
BLM<br>(NM_000057.3)	***CPA1***<br>(NM_001868.3)	***FANCE***<br>(NM_021922.2)	***KIT***<br>(NM_000222.2)	***PALB2***<br>(NM_024675.3)	***RECQL***<br>(NM_002907.3)	***RPS7***<br>(NM_001011.3)	***TP53***<br>(NM_000546.5)
BMPR1A<br>(NM_004329.2)	***CTNNA1***<br>(NM_001903.3)	***FANCF***<br>(NM_022725.3)	***LZTR1***<br>(NM_006767.3)	***PALLD***<br>(NM_001166110.1)	***RECQL4***<br>(NM_004260.3)	***RUNX1***<br>(NM_001754.4)	***TSC1***<br>(NM_000368.4)
BRCA1<br>(NM_007294.3)	***CTR9***<br>(NM_014633.4)	***FANCG***<br>(NM_004629.1)	***MAX***<br>(NM_002382.4)	***PDGFRA***<br>(NM_006206.4)	***REST***<br>(NM_005612.4)	***SDHA****<br>(NM_004168.3)	***TSC2***<br>(NM_000548.3)
BRCA2<br>(NM_000059.3)	***CTRC***<br>(NM_007272.2)	***FANCI***<br>(NM_001113378.1)	***MC1R***<br>(NM_002386.3)	***PHOX2B****<br>(NM_003924.3)	***RET***<br>(NM_020975.4)	***SDHAF2***<br>(NM_017841.2)	***VHL***<br>(NM_000551.3)
BRIP1<br>(NM_032043.2)	***DICER1***<br>(NM_177438.2)	***FANCL***<br>(NM_018062.3)	***MEN1***<br>(NM_130799.2)	***PIK3CA***<br>(NM_006218.2)	***RINT1***<br>(NM_021930.4)	***SDHB***<br>(NM_003000.2)	***WRN****<br>(NM_000553.4)
BUB1B<br>(NM_001211.5)	***DIS3L2***<br>(NM_152383.4)	***FANCM***<br>(NM_020937.2)	***MET***<br>(NM_001127500.1)	***PMS2***<br>(NM_000535.5)	***RNF43***<br>(NM_017763.5)	***SDHC***<br>(NM_003001.3)	***WT1***<br>(NM_024426.4)
CASR<br>(NM_000388.3)	***EGFR***<br>(NM_005228.3)	***FH***<br>(NM_000143.3)	***MITF****<br>(NM_000248.3)	***POLD1***<br>(NM_002691.3)	***RPL11***<br>(NM_000975.3)	***SDHD***<br>(NM_003002.3)	***XRCC2***<br>(NM_005431.1)

*Only sequencing analysis
